# Effects of the Coronavirus Job Retention Scheme (Furlough) on Lifestyle Health-Related Behaviors and Mental Health Symptoms in UK-Based Truck Drivers

**DOI:** 10.1097/JOM.0000000000003740

**Published:** 2026-04-21

**Authors:** Mohsen Sayyah, Nicola J. Paine, Aron P. Sherry, James A. King, Veronica Varela-Mato, Katharina Ruettger, Amber Guest, Daniel Longman, Colin N. Shaw, Stacy A. Clemes

**Affiliations:** 1 From the School of Sport, Exercise and Health Sciences, Loughborough University, Loughborough, UK (M.S., N.J.P., J.A.K., V.V.-M., A.G., D.L., S.A.C.); National Institute for Health Research (NIHR) Leicester Biomedical Research Centre, University Hospitals of Leicester NHS Trust and University of Leicester, Leicester, UK (N.J.P., J.A.K., S.A.C); Lifespan and Population Health, School of Medicine, University of Nottingham, Nottingham, UK (A.P.S.); Department of Sports and Health Sciences, Faculty of Human Science, University of Potsdam, Potsdam, Germany (K.R.); and Department of Anthropology, University of Zurich, Zurich, Switzerland (C.N.S.).

**Keywords:** COVID-19 lockdown, furloughs, health-related behavior, physical activity, sleep duration, mental health, truck drivers

## Abstract

**Objective:**

To compare lifestyle health-related behaviors and mental health symptoms between COVID-19 furloughed and non-furloughed UK-based truck drivers.

**Methods:**

91 Structured Health Intervention For Truckers-UK trial participants completed an online COVID-19-specific survey (May–July 2020); 44 (48%) were furloughed. Survey data were linked with prepandemic baseline and 6-month follow-up measures to examine longitudinal changes.

**Results:**

Furloughed drivers reported longer sleep, more frequent nature visits, longer standing time, and greater engagement in new physical activity. Longitudinally, furloughed drivers showed reduced anxiety during lockdown and increased nature visits. However, by 6-month follow-up, once work resumed, anxiety returned to baseline, engagement in new physical activity declined, and chronic fatigue increased.

**Conclusions:**

Furlough was associated with short-term positive health-related behaviors, but these gains were not sustained after drivers returned to work. Lasting health improvements are likely to require structural workplace changes and targeted interventions.

## Abstract 2

We found that when work demands were lifted under the furlough scheme during the COVID-19 lockdown, commercial drivers adopted healthier behaviors. These findings demonstrate that upstream policy change and structural workplace interventions are essential for reducing fatigue-related risk and supporting sustained improvements in health behaviors within this underserved high-risk workforce.

## Graphical abstract

**Figure:**
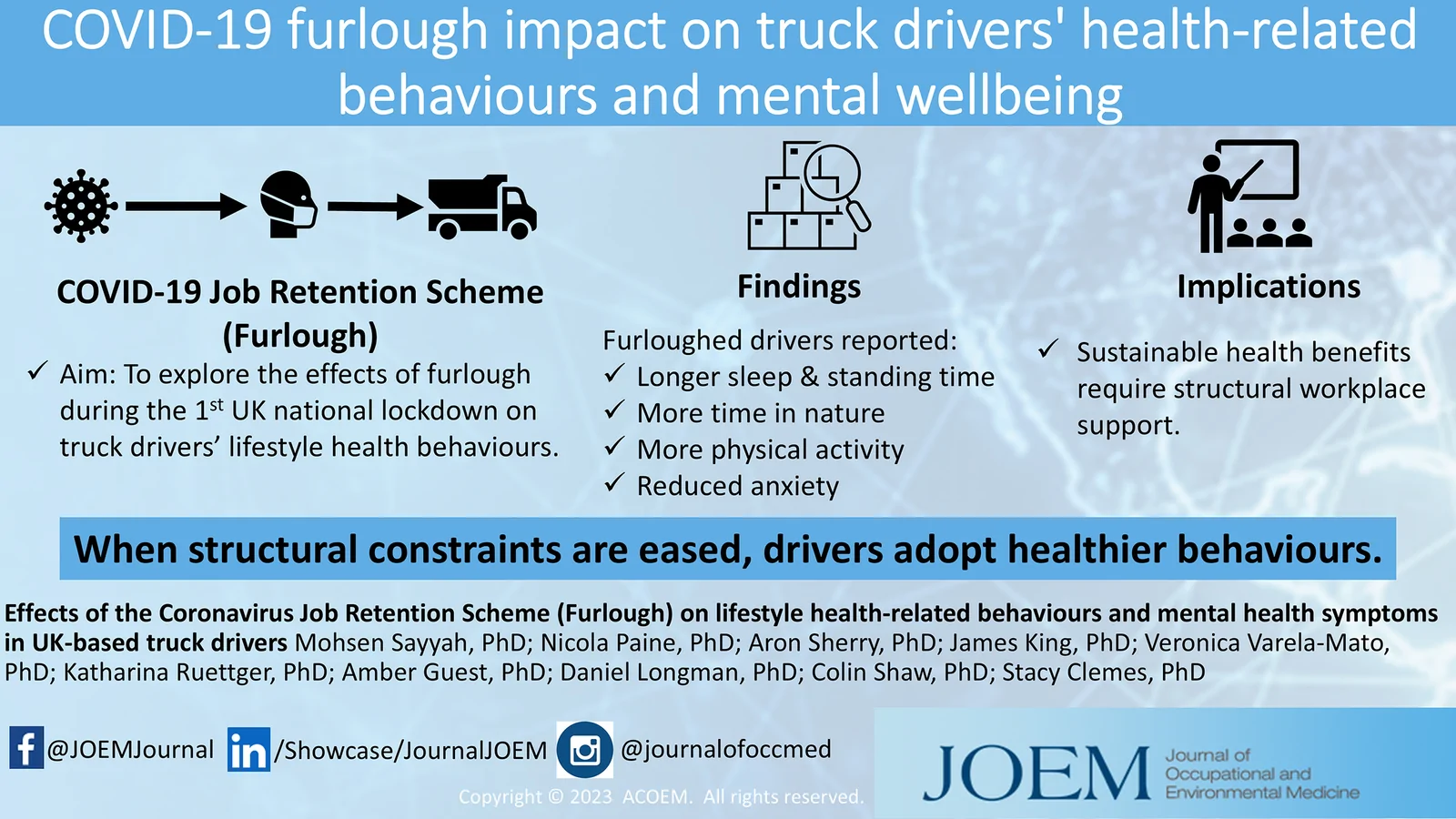

**LEARNING OUTCOMES**

- UK truck drivers on furlough slept more, spent more time in nature, and were more active.
- Health benefits diminished once furloughed drivers returned to work.
- Lasting health benefits require structural workplace changes and targeted interventions.

The spread of the SARS-CoV-2 virus led the UK government to introduce unprecedented measures such as social distancing, isolation, and movement restrictions. In March 2020, a national lockdown was announced to slow the spread of the disease and to protect the National Health Services ability to cope and save lives. To support employers whose operations were severely impacted, the UK government launched the Coronavirus Job Retention Scheme (furlough), which allowed employers to retain staff on temporary leave while covering 80% of their wages up to £2500/mo. By the end of May 2020, 8.7 million workers had been furloughed in the UK.^1^

Emerging research suggests furlough may have reduced COVID-19 exposure risks early in the pandemic. For example, Gittins et al^2^ found lower infection rates among furloughed UK workers. Beyond infection risk, furlough has also been linked to mental health outcomes, with Esteve-Matalí et al^3^ reporting reduced mental health problems, although effects varied by sex, occupation, and employment conditions.

Large-scale UK studies also suggest furlough may yield mixed effects on health behaviors. In the general population, it was associated with improved sleep and physical activity.^4^ Among hospitality workers, however, Grandey et al^5^ found that furlough led to both increased job threat and more time for recovery from work demand (eg, rest and leisure), supporting a dual-pathway model in which furlough simultaneously promoted opportunities that benefited health behaviors while also increasing psychological strain. This duality and occupational variation underscore the need to examine furlough effects in different occupational groups. In some contexts, furlough may offer recovery opportunities; in others, it may disrupt routine and exacerbate stress. The outcomes likely depend on occupational demands, job security, and social support. One such group notably underrepresented in furlough research is heavy goods vehicle drivers, referred to here as truck drivers, who are considered an at-risk occupational group with high levels of sedentary time, poor sleep, and elevated cardiometabolic and mental health risks.^6,7^

The COVID-19 pandemic had a well-documented detrimental impact on mental health both nationally and internationally with increases in anxiety, depression, and psychological distress widely reported.^8–10^ Commercial drivers, already prone to poor mental health and fatigue, may have been particularly vulnerable, making it especially important to examine whether furlough offered relief or introduced new challenges. Furthermore, there was a shortfall of 60,000 truck drivers^11^ in the UK in 2015, which increased to an estimated 100,000 in 2021^12^ owing to the combined effects of COVID-19 and Brexit. During the first UK lockdown, the Department for Transport temporarily relaxed EU drivers’ hours regulations, allowing those delivering essential goods to extend driving time up to 55 hours/wk,^13^ while others, particularly those in hospitality supply chains, were placed on furlough through the Government’s job retention scheme.

The high prevalence of mental health challenges and fatigue among commercial drivers underscores the importance of identifying potential protective factors. One such factor, increasingly recognized in occupational health research, is exposure to natural environments. Spending time in nature (including time in forests, parks, gardens, sports fields, allotments, woodlands, lakes/rivers, coastlines, or mountains) is associated with numerous physiological^14^ and psychological benefits,^15–17^ including reduced stress,^18^ improved cardiovascular health,^19^ and lower fatigue.^20^

For truck drivers, who face high levels of sedentary behavior, irregular sleep, and fatigue risk, nature exposure may be particularly valuable. Recent study showed that drivers visiting nature at least once per week reported substantially lower chronic and acute fatigue both before and during the COVID-19 pandemic.^20^ Given these established links, and the relevance of fatigue and mental health to the present study, time in nature may represent an important factor for supporting the health and wellbeing of truck drivers.

Considering the structural barriers to positive health-enhancing behaviors (eg, prolonged sitting, limited opportunities for activity, poor dietary options, shift work, and sleep restriction) experienced by truck drivers, we hypothesized that furlough, by temporarily relieving some of the barriers to health-enhancing behaviors, could facilitate short-term improvements in health behaviors and wellbeing. This hypothesis aligns with findings from the Structured Health Intervention For Truckers-UK (SHIFT-UK) randomized controlled trial,^7^ which evaluated the effectiveness of a multicomponent health promotion program among UK truck drivers. The intervention group showed improvements in physical activity, sedentary behaviour, and time spent in moderate-to-vigorous physical activity, particularly on nonworkdays.

These findings suggest that when structural constraints are eased, commercial drivers are both willing and able to adopt healthier behaviors. Building on this work, we sought to examine whether similar improvements could emerge in response to a naturally occurring, policy-driven disruption such as furlough.

The primary purpose of this study was to explore differences in lifestyle health-related behaviors and mental health symptoms between furloughed and non-furloughed truck drivers during and after the first UK COVID-19 lockdown.

## METHODS

### Procedure

After the easing of COVID-19 restrictions in England in May 2020, an online “COVID-19” survey (distributed by the “Online Surveys” platform) was administered between May and July 2020 to long-haul truck drivers participating in the “SHIFT-UK” trial; a randomized controlled trial evaluating the effectiveness and cost-effectiveness of a lifestyle-behavioral health intervention delivered within the workplace setting of a large logistics company.^21^

**FIGURE 1.**
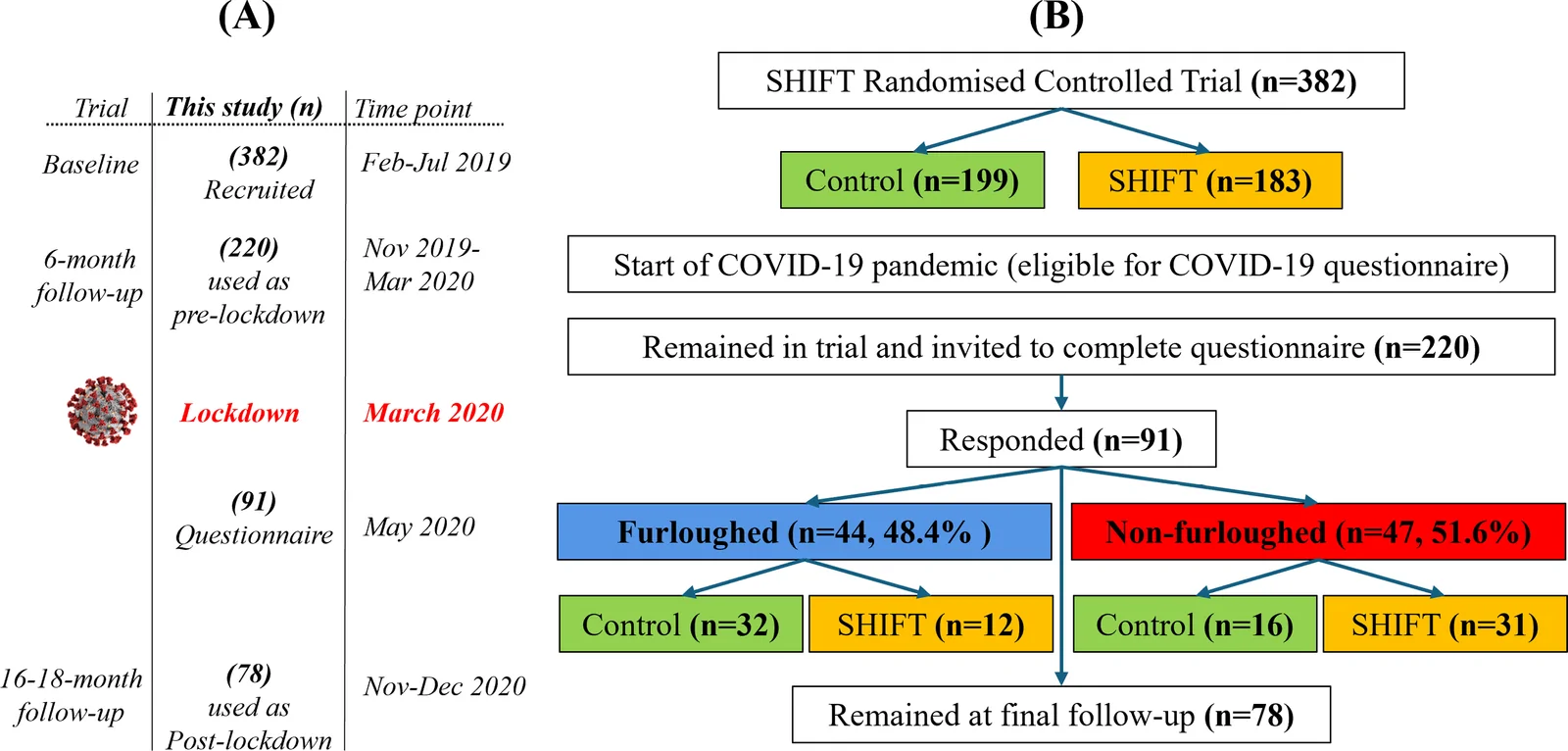
(A) Timeline of SHIFT-UK trial measurements and analytical timepoints used in the present study. (B) Participant flow from the SHIFT randomized controlled trial to the COVID-19 questionnaire and final follow-up, stratified by furlough status and trial arm (control vs. SHIFT).

Overall, 386 truck drivers were enrolled in the trial, recruited across 25 depots throughout the Midlands region of the UK, from our partner logistics organization. For the trial, baseline data were collected between February and July 2019, 6-month follow-up data between November 2019 and March 2020, and the 16–18-month (final) follow-up data between November and December 2020. The timeline of SHIFT-UK trial measurements is illustrated in Figure 1A.

For the present analysis, we included participants who completed the online COVID-19 survey and remained in the trial through final follow-up.^1^ Data collected at the 6-month trial follow-up (November 2019–March 2020), before the first UK COVID-19 lockdown, were treated as the prelockdown baseline for this study. Data collected at the final trial follow-up (November–December 2020), 6 months after the COVID-19 questionnaire, were treated as postlockdown follow-up.

This study is reported in accordance with the Strengthening the Reporting of Observational Studies in Epidemiology guidelines. The completed Strengthening the Reporting of Observational Studies in Epidemiology checklist is provided as Supplemental Digital Content 1, https://links.lww.com/JOM/C486.

Ethical approval for the SHIFT randomized controlled trial was granted by the Loughborough University Ethics Approvals (Human Participants) Sub-Committee (reference R17-P063), with subsequent approval obtained for the inclusion of the additional online COVID-19 survey (reference 2020-1444-1221). All participants provided written informed consent before participation, and the study was conducted in accordance with the Declaration of Helsinki.

### Measures

Participants reported their weight (kg), number of years worked as a truck driver, furlough status, average sleep duration (hours/d) in the past 14 days, alcohol intake frequency and quantity consumption (units/d) using questions 1 and 2 from the Alcohol Use Disorders Identification Test,^22^ sitting and standing times (hours/d), chronic and acute fatigue, inter-shift recovery score using the Occupational Fatigue Exhaustion Recovery (OFER) 15 Scale,^23^ frequency of spending time in nature and whether they had engaged in any new forms of physical activity because the COVID-19 lockdown (yes/no), with an optional follow-up open-text question inviting brief details (eg, apps, home workouts, or outdoor activities).

Symptoms of anxiety and depression were assessed using the Hospital Anxiety and Depression Scale (HADS).^24^ Body mass index (BMI) was estimated using participants’ height measured at baseline of the SHIFT trial and their self-reported weight in the COVID-19 questionnaire. Further details about all measures taken at baseline (prepandemic) are reported elsewhere.^21^

### Statistical Analysis

All analyses were conducted using IBM SPSS Statistics (version 29; IBM Corp, Armonk, NY). The Kolmogorov–Smirnov test assessed the normality of continuous variables. Descriptive statistics were calculated for all demographic and behavioral variables. Between-group comparisons (furloughed vs. non-furloughed drivers) used Chi-squared tests for categorical variables. For continuous data, independent- and paired-sample *t* tests were used for normally distributed variables, and Mann–Whitney *U* or Wilcoxon signed-rank tests were used for nonnormally distributed variables.

To examine predictors of key behavioral outcomes during lockdown (sleep duration and time spent in nature), hierarchical multiple regression analyses were conducted. These outcomes were selected because they showed significant between-group differences and are theoretically relevant to occupational recovery and health. In addition, although both anxiety and depression were considered as psychological predictors, depression was excluded from the final regression models owing to high collinearity with anxiety (*r* = 0.73), lack of statistical significance (*P* = 0.913), and no added explanatory power (Δ*R*^2^ = 0.000). Anxiety was retained based on its stronger theoretical and empirical association with sleep disruption during the COVID-19 lockdown. Therefore, 3 models were run for each outcome, Model 1 included furlough status (coded as 1 = furloughed, 0 = non-furloughed) and covariates (age, sex, education, ethnicity, and years working as a truck driver), Model 2 added anxiety scores (from HADS) to assess mental health contributions, and Model 3 added interaction between trial arm (intervention vs. control) and furlough status to evaluate whether intervention had influenced furloughed and non-furloughed drivers.

For longitudinal within-group comparisons (prepandemic, during lockdown, and 6-month postlockdown), Friedman tests were conducted separately for furloughed and non-furloughed participants to assess changes in anxiety, inter-shift recovery, chronic fatigue, and nature visit frequency. Post hoc Wilcoxon signed-rank tests were used when significant differences were found, with Bonferroni correction applied to adjust for multiple comparisons. To facilitate visual comparison of trends across outcomes with different scales and units, raw scores for anxiety, inter-shift recovery, and chronic fatigue were standardized to *Z*-scores (mean = 0, standard deviation [SD] = 1) before plotting figures. This transformation preserved the shape of change over time but removed the original units. All statistical analyses were conducted on the original, unstandardised values.

## RESULTS

Of the 220 participants who remained in the trial at the start of the pandemic, 91 (41% response rate from those invited, mean age: 51.3 ± 9 years, BMI: 29.5 ± 4.7 kg/m^2^, 98.9% male) completed the COVID-19 questionnaire. Table 1 presents prelockdown characteristics measured at the 6-month SHIFT-UK follow-up (November 2019–March 2020), stratified by subsequent furlough status. Most characteristics were comparable between groups; however, trial arm allocation was statistically associated with furlough status (χ^2^(1) = 13.65, *P* < 0.001). Participant characteristics and descriptive statistics summarizing lifestyle behaviors and mental health symptoms during the first UK national lockdown are presented in Table 2.

**TABLE 1.** Participant’s Characteristics of the Study Sample Stratified Between Furloughed and Non-furloughed Drivers Measured at the 6-Month SHIFT-UK Follow-up (November 2019–March 2020), Before First UK COVID-19 Lockdown.

	Full Sample	Furlough	Non-furlough	P
Participants	91 (98.9% male)	44 (48.4%)	47 (51.6%)	
Trial arms				**<0.001** ^a^
Intervention	43 (47.3%)	12 (13.2%)	31 (34.1%)	
Control	48 (52.7%)	32 (35.2%)	16 (17.6%)	
Ethnicity				0.964^a^
White	86 (95.6%)	42 (46.7%)	44 (48.9%)	
Others	4 (4.40%)	2 (2.2%)	2 (2.2.%)	
Truck duration, y	16	15	19	0.437^b^
Age	51.3 ± 9.2	52.0 ± 8.6	50.6 ± 9.7	0.462^c^
Weight, kg	90.8 (82.6, 99.4)	90.0 (83.8, 110.0)	90.9 (80.7, 99.0)	0.601^b^
BMI, kg/m^2^	28.7 (26.7, 31.7)	29.1 (26.8, 32.5)	28.2 (26.5, 31.2)	0.349^b^
Education				0.763^a^
GCSEs	70 (77.8%)	31 (34.4%)	39 (43.3%)	
A-levels	9 (10.0%)	5 (5.6%)	4 (4.4%)	
University graduate	3 (3.3)	2 (2.2%)	1 (1.1%)	
Master’s degree	2 (2.2%)	1 (1.1%)	1 (1.1 %)	
Other	6 (6.7%)	4 (4.4%)	2 (2.2%)	

Values are presented as n (%), mean ± SD, or median (IQR).

a Denotes the findings from Chi-squared tests.

b *P* values denote the findings from Mann–Whitney *U* tests.

c *P* value denotes the results of the independent samples *t* test.

**TABLE 2.** Descriptive Statistics Stratified by Furlough Status at the Time of Completing the COVID-19 Questionnaire

	Full Sample	Furlough	Non-furlough	P
Anxiety (HADS score 0–21)^a^	3.0 (1.0, 6.0)	2.0 (1.0, 5.7)	4.0 (1.0, 6.0)	0.271^f^
No symptoms	80 (87.9%)	40 (44.0%)	40 (44.0%)	
Mild	4 (4.4%)	2 (2.2%)	2 (2.2%)	
Moderate	4 (4.4%)	1 (1.1%)	3 (3.3%)	
Severe	3 (3.3%)	1 (1.1%)	2 (2.2%)	
Depression (HADS score)^a^	1.0 (0.0, 5.0)	1.5 (0.0, 5.00)	1.0 (1.0, 5.0)	0.734^f^
No symptoms	74 (81.3%)	38 (41.8%)	36 (39.6%)	
Mild	13 (14.3%)	5 (5.5%)	8 (8.8%)	
Moderate	2 (2.2%)	1 (1.1%)	1 (1.1%)	
Severe	2 (2.2%)	0	2 (2.2%)	
Alcohol frequency (5-point scale)^b^	3 (2.0, 4.0)	3 (3.0, 4.0)	3 (2.0, 4.0)	0.198^f^
Alcohol unit consumption^b^	2 (1.0, 3.0)	2 (1.0, 3.0)	2 (2.0, 4.0)	0.202^f^
OFER-15 chronic fatigue score^c^	32.7 ± 27.1	25.5 ± 23.9	36.3 ± 28.1	0.114^g^
OFER-15 acute fatigue score^c^	46.7 (20.0, 63.3)	41.7 (26.6, 69.6)	46.7 (16.6, 66.6)	0.756^f^
OFER-15 inter-shift recovery score^c^	56.6 (43.3,83.3)	58.3 (34.2, 82.5)	56.6 (46.6, 83.3)	0.626^f^
Sitting time, h/d	6.9 ± 2.5	7.1 ± 2.6	6.7 ± 2.4	0.450^g^
Standing time, h/d	1.0 (1.0, 2.0)	2.0 (1.0, 2.0)	1.0 (1.0, 2.0)	**0.036** ^f^
Work-related driving, h/d		0^e^	6.7	
Sleep duration increased^d^	25 (27.5%)	23 (25.3%)	2 (2.2%)	**<0.001** ^h^
Sleep duration, h/d	7.0 (6.0, 7.5)	7.0 (7.0, 8.5)	6.5 (6.0, 7.0)	**<0.001** ^f^
Frequency of nature visits, time/wk	2 (1.0, 3.0)	3.0 (2.0, 3.0)	2.0 (0.0, 3.0)	**0.005** ^f^
Never	21 (23.1%)	4 (4.4%)	17 (18.7%)	
Once/wk	10 (11.0%)	5.0 (5.5%)	5 (5.5%)	
2–3 times/wk	22 (24.2%)	10 (11.0%)	12 (13.2%)	
Almost everyday	24 (26.4%)	18 (19.8%)	6 (6.6%)	
Everyday	14 (15.4%)	7 (7.7%)	7 (7.7%)	
Engaged in NEW form of PA	23 (25.3%)	16 (17.6%)	7 (7.7%)	**0.019** ^h^

Values are presented as n (%), mean ± SD, or median (IQR), and (h/d) refers to hours per 24 hours cycle. Bold P values indicate statistically significant results (P < 0.05).

a HADS Anxiety and Depression scores range from 0 to 21, with higher scores indicating a greater degree of anxiety/depression, and classified as: 0–7 = no symptoms, 8–10 = mild, 11–14 = moderate, and 15–21 = severe.

b Alcohol frequency and unit consumption were based on items 1 and 2 of the Alcohol Use Disorders Identification Test (AUDIT).^23^

c Chronic fatigue, acute fatigue, and inter-shift recovery were measured using the OFER-15 (Occupational Fatigue Exhaustion Recovery) scale.^24^ Each subscale score ranges from 0 to 100, with higher scores indicating greater levels of fatigue or recovery.

d Based on a single question: “Since the COVID-19 restrictions, has the amount of time you spend sleeping changed?” followed by: “If yes, please indicate whether it increased or decreased.”

e On temporary leave under the Coronavirus Job Retention Scheme (furlough).

f *P* values denote the findings from Mann–Whitney *U* tests.

g *P* value denotes the results of the independent samples *t* test.

h Denotes the findings from Chi-squared tests.

A total of 44 participants (48.4% of respondents) reported being furloughed during the first UK national lockdown. Furloughed and non-furloughed drivers did not differ significantly in sex, age, weight, BMI, or years working as a truck driver. Furlough rates differed by trial arm, with 67% of participants in the control group (32 out of 48) and 28% in the intervention group (12 out of 43) reporting being furloughed (Fig. 1).

Baseline characteristics of participants included in the present analysis were broadly comparable to the wider SHIFT-UK cohort (Supplemental Digital Content 2, https://links.lww.com/JOM/C487). No statistically significant differences were observed for BMI, years driving as trucker, sleep duration, sleep efficiency, light physical activity, anxiety category, or depression category. A small but statistically significant difference was observed for age (median 51.0 vs. 49.0 years, *P* = 0.017), with participants included in this analysis being slightly older than the wider cohort. Seventy-eight of the 91 participants remained in the SHIFT-UK trial at final follow-up, and their data were used as the 6-month postlockdown measure for this study.

### Online COVID-19 Survey

As shown in Table 2, furloughed and non-furloughed drivers did not differ significantly during the first UK national lockdown in alcohol intake frequency and consumption, chronic and acute fatigue, inter-shift recovery, or self-reported sitting time. Sitting and standing time values reflect nonwork-related activities for furloughed drivers, as they reported zero driving hours during lockdown owing to being mandated off work. In contrast, for non-furloughed drivers, sitting time is largely attributable to time spent driving.

There were no significant differences in self-reported scores for symptoms of anxiety and depression between groups (both *P* > 0.05). Furloughed drivers reported an increase in their sleep (52% vs. 4%, *P* < 0.001) with significantly longer duration (7.0 vs. 6.5, hours/d, *P* < 0.001) over the past 14 days, a significantly higher frequency of spending time in nature (median = 3 vs. 2 times/wk, *P* < 0.01) and were significantly more likely to report trying a new form of physical activity (36% vs 15%, *P* < 0.05). The most common new forms of physical activity reported included: cycling, walking, gardening, running, weight training at home, boxing, exercise at home, and DIY.

### Changes Across Prelockdown, Duringlockdown, and Postlockdown Timepoints

There were no significant differences between furloughed and non-furloughed drivers in sitting time, standing time, depression, or fatigue (chronic or acute) at either prepandemic or lockdown timepoints.

**FIGURE 2.**
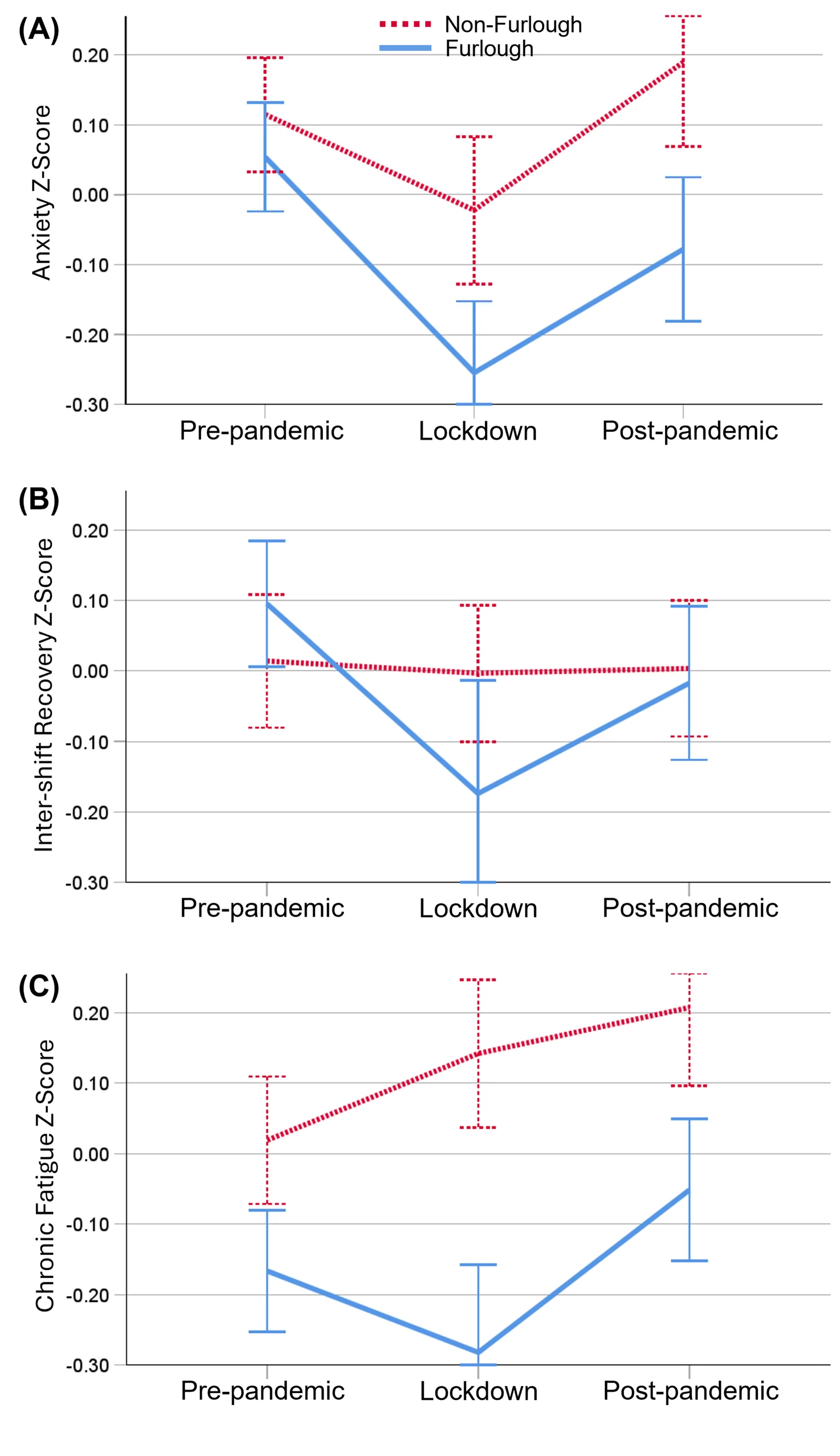
Standardized mean scores (*Z*-scores ± 95% confidence intervals) for (A) anxiety, (B) inter-shift recovery, and (C) chronic fatigue in furloughed (solid blue line) and non-furloughed (dashed red line) drivers before the COVID-19 pandemic, during the first UK lockdown, and 6 months post-lockdown. Each outcome was converted to a *Z*-score (mean = 0, SD = 1) to place all variables on a common scale, allowing visual comparison of change patterns over time rather than absolute values. The choice of solid and dashed lines, in addition to colour differences, ensures accessibility for readers with colour vision deficiencies.

Figure 2A presents changes in anxiety symptoms among commercial drivers measured at 3 timepoints: before the COVID-19 pandemic, during the first UK lockdown, and at a 6-month follow-up. A Friedman test revealed a statistically significant difference in anxiety scores over time among furloughed drivers (χ^2^(2) = 13.68, *P* = 0.001), but not among those who remained working throughout the pandemic (χ^2^(2) = 4.16, *P* = 0.125).

Post hoc Wilcoxon signed-rank tests indicated that, for furloughed drivers, anxiety symptoms significantly decreased from the prepandemic period to during the lockdown (*Z* = −2.59, *P* = 0.010). There were no differences in symptoms of anxiety between prepandemic and the postpandemic measures in these drivers (*Z* = −1.88, *P* = 0.060). In contrast, drivers who were not furloughed showed no significant changes in anxiety symptoms across the 3 timepoints.

Figure 2B displays mean inter-shift recovery scores across 3 timepoints, prelockdown, during the first UK COVID-19 lockdown, and 6-months postlockdown, stratified by furlough status. Note that furloughed drivers were not working shifts during lockdown. A Friedman test revealed no statistically significant difference in inter-shift recovery scores across the 3 timepoints for furloughed drivers (χ^2^(2) = 1.31, *P* = 0.521). However, exploratory post hoc Wilcoxon signed-rank tests indicated that scores were significantly lower during lockdown compared with prelockdown (*Z* = −2.36, *P* = 0.018), while no significant differences were found between pre and postlockdown (*P* = 0.597), or between lockdown and postlockdown (*P* = 0.506). For non-furloughed drivers, inter-shift recovery scores remained stable across all timepoints, with no statistically significant differences observed (*P* values >0.05).

Figure 2C displays changes in chronic fatigue scores at 3 timepoints. A Friedman test found no statistically significant differences in chronic fatigue scores across the 3 timepoints for either group (*P* > 0.05).

**FIGURE 3.**
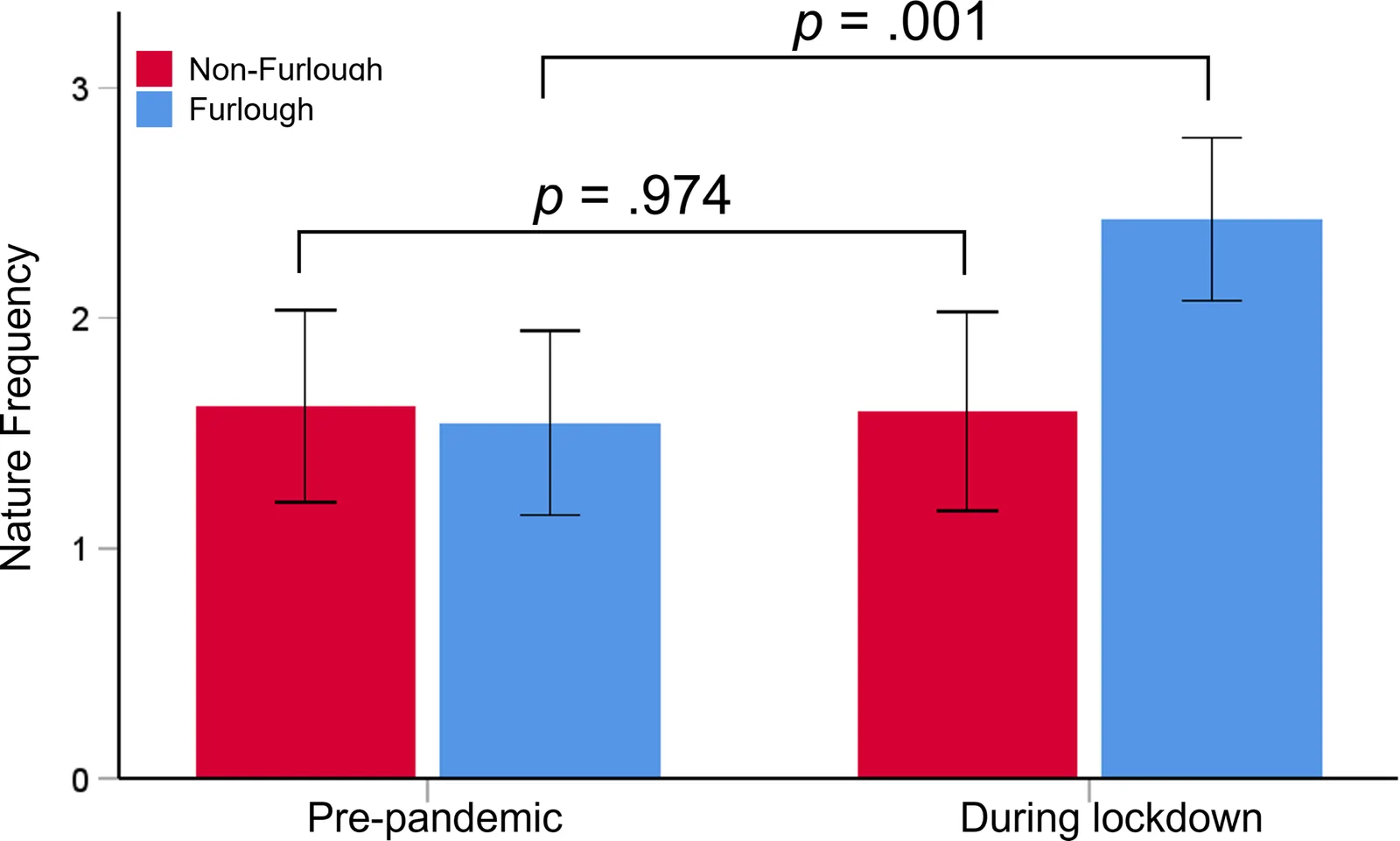
Frequency of nature visits before the COVID-19 pandemic and during the first UK lockdown among furloughed (blue) and non-furloughed (red) truck drivers. Note that no post-lockdown data were collected for this variable. A Wilcoxon Signed Ranks Test indicated a significant increase in nature visits among furloughed drivers (*P* = 0.001) but no change among non-furloughed drivers (*P* = 0.974). Error bars represent 95% confidence intervals.

As shown in Figure 3, furloughed drivers reported a significant increase in the frequency of spending time in nature during the first UK national lockdown compared with prepandemic levels (*P* = 0.001). In contrast, no significant change was observed among non-furloughed drivers (*P* = 0.974). During lockdown, 41% (18 of 44) of furloughed drivers reported visiting nature almost daily (≥4 days/wk), representing a rise from 7% (3 of 44) before the pandemic. No similar shift was observed in the non-furloughed group.

Table 3 presents the results of hierarchical multiple regression models predicting sleep duration and time in nature during the first UK COVID-19 lockdown, with *B*, β, and *P* values shown for all predictors.

**TABLE 3.** Summary of Hierarchical Multiple Regression Models Predicting Sleep Duration and Nature Visit Frequency During the First UK COVID-19 Lockdown

	*B*	β	*P*	Adjusted *R*^2^	∆*R*^2^	*F* Change	Sig. *F* Change
Sleep duration							
Model 1				0.175	0.243	30.570	**0.002**
Sex	−0.304	−0.027	0.790				
Age	0.009	0.061	0.636				
Ethnicity	−0.077	−0.108	0.282				
Education level	−0.033	−0.037	0.715				
Driving experience	−0.013	−0.107	0.419				
Trial arm (intervention/control)	−0.488	−0.199	**0.065**				
Furlough status	0.876	0.358	**0.002**				
Model 2				0.217	0.048	50.171	**0**.**026**
Sex	0.194	0.017	0.867				
Age	0.009	0.060	0.630				
Ethnicity	−0.079	−0.111	0.255				
Education level	−0.024	−0.027	0.789				
Driving experience	−0.016	−0.131	0.310				
Trial arm (intervention/control)	−0.538	−0.220	**0.038**				
Furlough status	0.811	0.331	**0.003**				
Anxiety	−0.072	−0.226	**0.026**				
Model 3				0.210	0.004	0.409	0.524
Sex	0.084	0.007	0.942				
Age	0.014	0.096	0.486				
Ethnicity	−0.082	−0.115	0.243				
Education level	−0.037	−0.041	0.689				
Driving experience	−0.017	−0.141	0.281				
Trial arm (intervention/control)	−0.362	−0.148	0.338				
Furlough status	0.978	0.399	**0.010**				
Anxiety	−0.067	−0.210	**0.045**				
Trial arm × furlough (interaction)	−0.362	−0.148	**0.338**				
Nature frequency							
Model 1				0.079	0.154	20.060	0.058
Sex	−10.584	−0.124	0.238				
Age	0.016	0.103	0.449				
Ethnicity	−0.039	−0.049	0.645				
Education level	0.014	0.014	0.894				
Driving experience	0.010	0.072	0.603				
Trial arm (intervention/control)	−0.249	−0.092	0.417				
Furlough status	0.731	0.269	**0.022**				
Model 2				0.071	0.003	0.308	0.581
Sex	−10.439	−0.113	0.295				
Age	0.016	0.103	0.450				
Ethnicity	−0.040	−0.050	0.637				
Education level	0.018	0.017	0.870				
Driving experience	0.009	0.067	0.635				
Trial arm (intervention/control)	−0.262	−0.096	0.396				
Furlough status	0.710	0.261	**0.027**				
Anxiety	−0.021	−0.060	0.581				
Model 3				0.069	0.008	0.778	0.380
Sex	−10.622	−0.127	0.244				
Age	0.025	0.156	0.296				
Ethnicity	−0.044	−0.056	0.600				
Education level	−0.004	−0.004	0.974				
Driving experience	0.007	0.052	0.713				
Trial arm (intervention/control)	0.030	0.011	0.947				
Furlough status	0.988	0.363	**0.030**				
Anxiety	−0.013	−0.035	0.750				
Trial arm × furlough (interaction)	−0.624	−0.158	0.380				

All models controlled for age, sex, education level, ethnicity, and years of driving experience. Model 1 included furlough status and demographic covariates. Model 2 added anxiety score. Model 3 added interaction between trial arms and furlough status. Δ*R*^2^ represents the change in explained variance from the previous model. Statistically significant values are reported in bold.

For sleep duration, Model 1 explained 17.5% of the variance (adjusted *R*^2^ = 0.175, *P* = 0.002), with furlough status being a strong and significant predictor (*B* = 0.876, β = 0.358, *P* = 0.002). Trial arm was not statistically significant at this stage (*P* = 0.065). In Model 2, the inclusion of anxiety significantly improved model fit (∆*R*^2^ = 0.048, *P* = 0.026). Anxiety emerged as a significant negative predictor of sleep duration (*B* = −0.072, β = −0.226, *P* = 0.026), indicating that higher anxiety was associated with shorter sleep. Trial arm also became a significant predictor (*B* = −0.538, β = −0.22, *P* = 0.038), suggesting that participants in the intervention arm reported shorter sleep duration during lockdown compared with those in the control arm. Model 3 introduced the interaction between trial arm and furlough status, but this term was not statistically significant (*B* = −0.375, β = −0.106, *P* = 0.524) and did not improve model fit (∆*R*^2^ = 0.004, *P* = 0.524). This suggests that the effect of furlough on sleep duration did not differ between trial arms.

For nature frequency, Model 1 explained 7.9% of the variance (Adjusted *R*^2^ = 0.079, *P* = 0.058), with furlough status emerging as the only significant predictor (*B* = 0.73, β = 0.27, *P* = 0.022). This indicates that furloughed drivers reported more frequent exposure to nature during lockdown than those who continued working. None of the demographic covariates or trial arm were significant in this model. Model 2 included anxiety, but it did not significantly contribute to model fit (∆*R*^2^ = 0.003, *P* = 0.581) and was not a significant predictor (*P* = 0.581). Model 3 added the interaction between intervention group and furlough status, which was also nonsignificant (*B* = −0.62, β = −0.16, *P* = 0.380), indicating that the effect of furlough on nature frequency did not vary by trial arm. Importantly, furlough status remained a significant predictor across all 3 models, highlighting a consistent association between being furloughed and greater frequency of nature visits.

In addition to these outcomes, analyses showed that furlough status was not significantly associated with, sitting time (*B* = 0.15, *P* = 0.800), alcohol intake frequency (*B* = 0.26, *P* = 0.265), alcohol unit consumption (*B* = −0.42, *P* = 0.205), symptoms of anxiety (*B* = −0.76, *P* = 0.383) or depression (*B* = −0.25, *P* = 0.779), body weight (*B* = 5.25, *P* = 0.114) or BMI (*B* = 1.84, *P* = 0.089), although the latter showed a trend toward significance.

None of the demographic covariates (age, sex, ethnicity, education, and driving experience) were significant in any model.

Overall, these findings suggest that furlough status was a consistent and significant predictor of longer sleep duration and more frequent nature visits. Anxiety symptoms independently predicted shorter sleep.

### Six-Month Postlockdown

Wilcoxon signed-rank tests were used to examine within-group changes from lockdown to the final follow-up (6-month post-lockdown), stratified by furlough status (Supplemental Digital Content 3, https://links.lww.com/JOM/C488). Among non-furloughed drivers, BMI (*Z* = −3.03, *P* = 0.002) and body weight (*Z* = −2.92, *P* = 0.003) increased significantly from lockdown to follow-up, and anxiety scores also increased (*Z* = −2.21, *P* = 0.027). No significant changes were observed for alcohol intake frequency, alcohol units, acute fatigue, or depression scores (all *P* > 0.05). Among furloughed drivers, no significant within-group changes were observed for BMI, body weight, alcohol intake, acute fatigue, anxiety, or depression (all *P* > 0.05), although depression showed a nonsignificant trend (*P* = 0.069). A generalized estimating equation model indicated that, averaged across lockdown and 6-month follow-up, furloughed drivers were more likely to report engagement in a new form of physical activity compared with non-furloughed drivers (Wald χ^2^ = 3.96, *P* = 0.047). However, there was no significant change over time and no group × time interaction (Supplemental Digital Content 4, https://links.lww.com/JOM/C489).

## DISCUSSION

This study is the first to examine how the UK’s Coronavirus Job Retention Scheme (furlough) affected lifestyle behaviors and mental health among truck drivers across 3 time points (prelockdown, duringlockdown, and 6-months postlockdown). During the first national lockdown (March–July 2020), furloughed drivers reported longer sleep, more frequent visits to nature, greater standing time, and higher engagement in new forms of physical activity compared with those who continued working. These findings provide novel insight into how temporary relief from occupational demands may enable positive behavioral changes in an at-risk workforce.

There were no statistically significant differences in anxiety, depression, or alcohol consumption between furloughed and non-furloughed drivers. Based on HADS severity categories, most participants reported no anxiety symptoms during lockdown (furloughed: 91%; non-furloughed: 85%), with mild to severe symptoms more common in non-furloughed drivers, although not significant. Furloughed drivers showed significantly lower anxiety scores and higher inter-shift recovery scores during lockdown compared with non-furloughed drivers, which may reflect general rest and recuperation in the absence of regular shift work. These findings are notable given that anxiety among truck drivers is typically higher than the national average and has been linked to a 4-fold increase in crash risk.^6^ Anxiety symptoms decreased during lockdown in furloughed drivers, suggesting a short-term psychological reprieve from work-related pressures, but returned to prepandemic levels by 6 months. In contrast, non-furloughed drivers showed no change. Engagement in new physical activity also declined postlockdown in the furloughed group (from 29.2% to 11.3%) but increased slightly in the non-furloughed group (14.9%–17.0%), possibly reflecting postlockdown fatigue or time pressures. These patterns suggest that while furlough may offer temporary benefits for mental health and behavior, sustaining these gains likely requires ongoing workplace support, including strategies to reduce fatigue and enable health-promoting routines.

Inter-shift recovery scores declined among furloughed drivers during lockdown, despite their absence from work. This finding may reflect pandemic-related stress, uncertainty, and disrupted routines, alongside the loss of structured work patterns and workplace social interaction. Importantly, the OFER scale captures perceived daily recovery and coping rather than recovery from actual shifts, which may explain why recovery did not improve despite reduced occupational demands. However, recovery scores returned to baseline by 6 months, suggesting readjustment as work resumed. In contrast, non-furloughed drivers showed no change, indicating stable but suboptimal recovery throughout the pandemic. These results challenge the assumption that time away from work automatically improves recovery and highlight that recovery depends not only on workload but also on structured routines, daily predictability, and social support, elements often disrupted during lockdown. The isolated nature of truck driving may further compound these challenges, underscoring the need for recovery-supportive environments that also promote social connection.^25–29^

We found that furloughed drivers visited nature significantly more often during lockdown, with 41% reporting visits ≥4 times/wk versus only 7% before the pandemic. In a separate analysis using the same cohort,^20^ increased nature exposure was associated with lower chronic and acute fatigue in truck drivers,^20^ a critical concern given that fatigue-related accidents in UK truck drivers^30^ stem not only from insufficient sleep but also from occupational factors such as long hours, irregular shifts, and driving demands. Nature-based interventions are an accessible, low-cost, and medication-free approach to improving health in this underserved population and should be considered in future occupational health strategies.

We hypothesized that the furlough scheme could lead to sustained health benefits if behavioral improvements persisted at 6 months postlockdown. Our findings partially support this: furloughed drivers reported longer sleep, more frequent nature visits, and greater engagement in new physical activities during lockdown, but participation declined sharply once work resumed. Regression models showed that furlough status was a strong predictor of longer sleep, whereas higher anxiety predicted shorter sleep, although most scores fell within the normal range, suggesting subclinical variation rather than clinical concern. Some under-reporting, particularly among working-class men, may also have influenced self-reported mental health.^31,32^ These results suggest that temporary relief from shift-related pressures allowed furloughed drivers to adopt healthier behaviors, yet sustaining these benefits likely requires structural workplace support that reduces time pressure, protects recovery, and promotes access to restorative environments. The absence of shift work and rigid schedules, combined with greater time outdoors, may have enabled more natural sleep-wake cycles, patterns that could be replicated through tailored occupational health programs.

Furloughed drivers reported higher sitting and standing times; however, this must be interpreted with caution. Among furloughed drivers, these behaviors were not occupationally driven (ie, not owing to driving or work duties), whereas in non-furloughed drivers, sitting time largely reflected work-related driving. It is also possible that nature visits during lockdown involved more passive activities (eg, sitting outdoors or spending time in gardens) rather than purposeful movement. This distinction may explain why furloughed drivers reported higher sitting and standing times yet remained largely inactive in leisure contexts, underscoring the need for behavioral interventions that promote active engagement outside of work.

An additional notable finding was the significant increase in BMI, body weight, and anxiety scores among non-furloughed drivers between lockdown and 6-month follow-up, whereas no comparable changes were observed among furloughed drivers. Although the magnitude of these changes was modest, they may reflect the occupational pressures faced by drivers who continued working throughout and immediately after lockdown. Unlike furloughed drivers, non-furloughed drivers remained exposed to the structural constraints of long-haul driving, including irregular shifts, prolonged sedentary time, and limited access to healthy food options. As restrictions eased and traffic volumes increased, work demands and road congestion may also have intensified, potentially contributing to elevated stress and anxiety levels. While causality cannot be inferred, these findings suggest that continued occupational exposure during the pandemic recovery phase may have had short-term adverse effects on physical and psychological health in drivers who remained working.

The findings from this study align closely with the broader program of work aimed at improving the health of commercial drivers through occupationally embedded interventions. In the SHIFT-UK trial,^7^ drivers showed the most notable lifestyle improvements on nonworkdays, when they had greater autonomy and relief from job-related constraints. Similarly, furlough created a temporary window for positive change, offering time, space, and a pause from shift-related pressures. Together, these studies illustrate 2 complementary pathways to health improvement: one through an unplanned policy disruption, and the other via a structured, scalable intervention. Both reinforce the idea that when occupational pressures are reduced and drivers are given appropriate support, they are willing and able to make meaningful health behaviour changes. The consistency in improvements across sleep, physical activity, and nature engagement strengthens the case for scaling workplace interventions that reflect the lived realities of this workforce. In this context, the SHIFT-UK program^33^ offers a practical vehicle for sustaining such gains, particularly in environments where discretionary time is limited.

Fatigue is a pervasive sense of reduced energy not solely caused by exertion and is closely linked to both physiological and psychological stressors, including heavy workloads, long hours, job strain, shift work, disturbed sleep, and poor overall health.^34–39^ These occupational constraints can lead to sustained psycho-physiological arousal, contributing to serious levels of fatigue,^40–43^ a higher prevalence of mental health conditions,^44^ and an increased risk of road accidents among commercial drivers.^45^ In our study, chronic fatigue significantly increased in furloughed drivers once they returned to work, suggesting that working conditions were a major driver of this change, consistent with prior evidence linking poor work environments to fatigue in truck drivers.^30^ Reducing fatigue in this workforce requires organizational measures such as optimized schedules, limits on consecutive shifts, and sufficient recovery opportunities, rather than reliance on individual coping alone.

In the workplace more broadly, there is mixed evidence on the health impacts of furlough during the COVID-19 pandemic, with outcomes varying by context, sector, and the design of furlough schemes. Some studies report negative effects, including worsening health among US healthcare workers,^46^ declines in wellbeing among furloughed or unemployed individuals in Spain^47^ and US hospitality workers,^48^ and increased non-COVID hospitalizations and deaths in US nursing homes after infection-related staff furloughs.^49^ In contrast, studies from the UK and Sweden found that well-structured, secure furlough schemes were associated with improved sleep, greater physical activity, and stable mental health^2,4,50^ with no increased risk of smoking or excessive drinking.^51^ Our findings align with this latter pattern, suggesting that perceived income stability and relief from work pressures may underpin short-term behavioral gains.

Variations in findings across studies may stem from inconsistent definitions of furlough and differing financial or psychological contexts. Some research found no mental-health impact,^52^ others reported benefits only for paid leave^53^ and some observed increased depression where furlough involved income loss.^54^ Importantly, job insecurity and poor organizational communication have also been linked to longer-term psychological strain.^3,5,55^ These insights highlight the need to clearly define furlough type and consider perceived job stability when evaluating psychological outcomes.

Although workplace stress and low job control are often linked to increased alcohol use,^56^ and some furlough studies report higher drinking,^57,58^ our truck driver sample showed no differences between furloughed and non-furloughed groups. Nationally, risky drinking rose from 25% pre-pandemic to 38% during the first lockdown, with 20%–33% of adults reporting increased.^59^ UK-representative data further suggest that higher pandemic-era drinking was driven by coping and enhancement motives, particularly among those with lower job security or poorer mental health.^60^ In contrast, truck drivers in our study appeared to maintain stable consumption, possibly owing to occupational compliance pressures and limited social drinking opportunities.

Compared with other occupational groups, truck drivers have a significantly lower life expectancy,^61,62^ largely owing to working conditions that limit opportunities to engage in healthy lifestyle behaviors.^63–65^ Factors such as sleep deprivation, psychological stress, long working hours, irregular shift patterns, and pressure to meet delivery schedules have all been reported as contributors to poor health in this group.^66^ These reinforce the view that the workplace environment plays a vital role in shaping the health of truck drivers.^6^ The present study adds to this evidence by showing that when these constraints were temporarily removed, drivers demonstrated capacity and motivation to improve their wellbeing. Future workplace strategies should therefore focus on reducing occupational fatigue through predictable schedules, promoting active breaks and access to nature, supporting adequate sleep, and embedding structured programs such as SHIFT-UK to sustain behavioral gains over time.

### Limitations

While our study offers unique and valuable insights into behavioral and mental health outcomes among furloughed truck drivers, several limitations should be acknowledged.

First, the sample was relatively small (n = 91), predominantly male and White British, reflecting both the demographic composition of the UK heavy goods vehicle driver workforce and the wider UK transport industry, which remains a male-dominated occupation. While this limits generalizability to more diverse populations, it accurately represents the occupational context under study and aligns with the study’s aim to examine furlough effects within this specific workforce.

Second, information on participants’ socioeconomic status, nativity or immigration background, and disability status was not collected as part of the original SHIFT trial dataset. However, given that all participants were employed by the same large UK logistics company, the cohort is likely to represent a relatively homogeneous working-class occupational group.

Third, furlough rates differed substantially by trial arm, with 67% of participants in the control group (32/48) and 28% in the intervention group (12/43) reporting being furloughed. The SHIFT trial was cluster-randomized in 2017 across 25 transport depots,^7^ whereas the COVID-19 pandemic and associated furlough decisions occurred in 2020, approximately 3 years later. Furlough status was determined independently by individual depots in response to operational demands during the pandemic and was not influenced by trial allocation. The observed imbalance therefore likely reflects depot-level variation in pandemic response rather than any effect of the intervention itself. Nonetheless, this uneven distribution limited our ability to directly compare furlough effects between trial arms.

Fourth, all behavioral, lifestyle, and mental health measures were self-reported, introducing the potential for recall and social desirability bias. For example, participants may have under-reported symptoms of anxiety or depression, which were low in our sample compared with what is typically observed in other high-risk occupational groups. This may reflect stigma around mental health, especially among male-dominated workforces such as truck drivers, or limitations in symptom recognition.

Finally, we did not explore participants’ subjective experiences of furlough, including perceptions of communication, fairness, or job security. Qualitative research by Szulc and Smith^67^ has shown that such perceptions can influence individuals’ sense of ability, motivation, and opportunity, factors that may shape behavioral responses to furlough.

## CONCLUSIONS

This study demonstrates that furloughed truck drivers made more positive lifestyle changes than their non-furloughed counterparts during the first UK COVID-19 lockdown (May–July 2020), including increased sleep duration, greater non-work-related standing time, more frequent nature exposure, and engagement in new forms of physical activity. These behaviors were accompanied by a temporary reduction in anxiety and represent lifestyle patterns typically associated with short-term health benefits. However, several benefits diminished after returning to work, with chronic fatigue significantly increasing postlockdown. The findings highlight both the potential for health improvements when occupational pressures are reduced and the difficulty of sustaining these gains without continued support. Furlough may provide a temporary reprieve that enables healthier behaviors, but lasting improvements in driver health will likely require structural workplace changes and targeted interventions.

## ACKNOWLEDGMENTS

We gratefully acknowledge the truck drivers who participated in the SHIFT project and completed the COVID-19 survey. Their time and insights made this research possible. S.A.C., J.A.K., and N.J.P. are also supported by the NIHR Leicester Biomedical Research Centre (Lifestyle theme). The views expressed are those of the authors and not necessarily those of the NHS, NIHR, or the Department of Health and Social Care.

## Supplementary Material

## Footnotes

1 Abstract submitted to conference proceedings in 2021.
