# Supplementary material for: Effects of the Coronavirus Job Retention Scheme (Furlough) on Lifestyle Health-Related Behaviors and Mental Health Symptoms in UK-Based Truck Drivers
Source: J Occup Environ Med. 2026 Apr 21;68(10):e668–78. doi: 10.1097/JOM.0000000000003740 (PMC13600360; doi:10.1097/JOM.0000000000003740)
Supplement: Supplementary file 2 [file joem-68-e668-s002.pdf]

SDC 2: Baseline characteristics of participants included in the present study compared with the wider SHIFT-UK Cohort, eligible for inclusion in these analyses.

**Variable**	**SHIFT-UK Main Trial (n=295) [b]**	**This study (n=91)**	**p-value [c]**
Sex (male)	291 (98.6%)	88 (96.7%)	
age	49.00	51.00	***.017***
BMI	29.9	29.4	*.334*
Truck duration (years)	16.0	16.0	*.346*
Sleep duration	5.55	5.84	*.093*
Sleep Efficiency	77.24	78.18	*.225*
Light Physical Activity (min/day)	67.90	65.74	*.978*
Anxiety (HADS Score 0-21) [a]			*.616*
No symptoms	229 (77.6%)	72 (79.1%)	
Mild	45 (16.7%)	15 (5.08%)	
Moderate	14 (4.7%)	2 (2.2%)	
Severe	2 (0.67%)	0	
Depression (HADS Score) [a]			*.184*
No symptoms	215 (72.8%)	71 (78%)	
Mild	40 (13.5%)	10 (10.9%)	
Moderate	9 (3.05%)	0	
Severe	0	0	

Note: Values are presented as n (%), median (IQR), and (min/day) refers to minutes per 24h cycle.

[a] HADS Anxiety and Depression scores range from 0 to 21, with higher scores indicating a greater degree of anxiety/depression, and classified as: 0-7 = no symptoms, 8-10 = mild, 11-14 = moderate, and 15-21 = severe.

[b] the SHIFT-UK trial cohort (n = 295) refers to participants enrolled in the main cluster-randomised trial phase relevant to the present analysis. Participants from the earlier pilot (pre-post) phase had completed follow-up prior to the onset of the COVID-19 pandemic and were therefore not eligible for inclusion in these analyses.

[c] Continuous variables were compared using Mann–Whitney U tests. Categorical variables were compared using χ^2 or Fisher–Freeman–Halton exact tests where appropriate.
