# Supplementary material for: Effects of the Coronavirus Job Retention Scheme (Furlough) on Lifestyle Health-Related Behaviors and Mental Health Symptoms in UK-Based Truck Drivers
Source: J Occup Environ Med. 2026 Apr 21;68(10):e668–78. doi: 10.1097/JOM.0000000000003740 (PMC13600360; doi:10.1097/JOM.0000000000003740)
Supplement: Supplementary file 3 [file joem-68-e668-s003.pdf]

SDC3: Within-group changes from lockdown to 6-month follow-up (final follow-up), stratified by furlough status

**Outcome**	**Non-furloughed (Lockdown)**	**Non-furloughed (Follow-up)**	**Paired N**	**Z**	**p**	**Furloughed (Lockdown)**	**Furloughed (Follow-up)**	**Paired N**	**Z**	**p**
**BMI (kg/m²)**	28.7 ± 4.4	29.0 ± 4.9	70	-3.03	**.002**	30.2 ± 4.9	30.2 ± 4.5	68	-0.72	.474
**Weight (kg)**	91.3 ± 12.9	93.8 ± 16.4	70	-2.92	**.003**	95.6 ± 15.4	95.5 ± 15.0	68	-0.61	.543
**Alcohol frequency (1–5)**	3.0 ± 0.9	3.6 ± 1.0	16	-0.37	.713	3.3 ± 1.1	3.6 ± 0.5	14	-1.61	.107
**Alcohol units (1–5)**	2.7 ± 1.3	2.4 ± 1.5	16	-0.74	.458	2.4 ± 1.4	3.3 ± 1.6	14	-0.28	.776
**Acute fatigue (OFER)**	45.9 ± 28.5	48.2 ± 26.5	82	-0.32	.748	43.9 ± 23.8	45.8 ± 25.0	42	-0.54	.586
**Anxiety (HADS)**	4.2 ± 3.9	5.0 ± 4.1	80	-2.21	**.027**	3.4 ± 3.7	4.1 ± 3.4	76	-1.57	.116
**Depression (HADS)**	3.5 ± 4.3	3.9 ± 4.4	80	-1.42	.155	3.0 ± 3.4	3.4 ± 3.1	76	-1.82	.069

Values are mean (SD). Within-group change tested using Wilcoxon signed-rank tests (two-tailed). “Paired N” reflects the number of participants with data at both lockdown and follow-up for that variable (varies due to missing follow-up data, particularly for alcohol measures).
