# Supplementary material for: Effects of the Coronavirus Job Retention Scheme (Furlough) on Lifestyle Health-Related Behaviors and Mental Health Symptoms in UK-Based Truck Drivers
Source: J Occup Environ Med. 2026 Apr 21;68(10):e668–78. doi: 10.1097/JOM.0000000000003740 (PMC13600360; doi:10.1097/JOM.0000000000003740)
Supplement: Supplementary file 4 [file joem-68-e668-s004.pdf]

SDC 4: Generalized estimating equation analysis of engagement in new physical activity from lockdown to 6-month follow-up.

**Effect**	**Wald χ^2**	**df**	**p-value**
**Furlough status**	3.96	1	**.047**
**Time**	0.16	1	.686
**Furlough x Time**	1.33	1	.249

Note: Generalized estimating equation model with binomial distribution and logit link. Exchangeable working correlation structure was specified. Time (lockdown vs 6-month follow-up) was treated as a repeated factor within participants.
